# Characterization of High-Value Bioactives in Some Selected Varieties of Pakistani Rice (*Oryza sativa* L.)

**DOI:** 10.3390/ijms13044608

**Published:** 2012-04-11

**Authors:** Muhammad Zubair, Farooq Anwar, Muhammad Ashraf, Md. Kamal Uddin

**Affiliations:** 1 Department of Chemistry and Biochemistry, University of Agriculture, Faisalabad-38040, Pakistan; E-Mail: zub474@yahoo.com; 2 Department of Chemistry, University of Sargodha, Sargodha-40100, Pakistan; 3 Department of Botany, University of Agriculture, Faisalabad-38040, Pakistan; E-Mail: ashrafbot@yahoo.com; 4 Institute of Tropical Agriculture, University Putra Malaysia-43400, Serdang, Selangor, Malaysia

**Keywords:** bioactives, Basmati rice, phytosterols, tocopherols, oryzanol, HPLC, GC-MS

## Abstract

The present study reports the composition and variation of fatty acids, sterols, tocopherols and γ-oryzanol among selected varieties namely Basmati Super, Basmati 515, Basmati 198, Basmati 385, Basmati 2000, Basmati 370, Basmati Pak, KSK-139, KS-282 and Irri-6 of Pakistani rice (*Oryza sativa* L). Oil content extracted with *n*-hexane from different varieties of brown rice seed (unpolished rice) ranged from 1.92% to 2.72%. Total fatty acid contents among rice varieties tested varied between 18240 and 25840 mg/kg brown rice seed. The rice tested mainly contained oleic (6841–10952 mg/kg) linoleic (5453–7874 mg/kg) and palmitic acid (3613–5489 mg/kg). The amounts of total phytosterols (GC and GC-MS analysis), with main contribution from β-sitosterol (445–656 mg/kg), campesterol (116–242 mg/kg), Δ^5^-avenasterol (89–178 mg/kg) and stigmasterol (75–180 mg/kg) were established to be 739.4 to 1330.4 mg/kg rice seed. The content of α-, γ- and δ-tocopherols as analyzed by HPLC varied from 39.0–76.1, 21.6–28.1 and 6.5–16.5 mg/kg rice seed, respectively. The amounts of different γ-oryzanol components (HPLC data), identified as cycloartenyl ferulate, 24-methylene cycloartanyl ferulate, campesteryl ferulate and β-sitosteryl ferulate, were in the range of 65.5–103.6, 140.2–183.1, 29.8–45.5 and 8.6–10.4 mg/kg rice seed, respectively. Overall, the concentration of these bioactives was higher in the Basmati rice cultivars showing their functional food superiority. In conclusion, the tested varieties of Pakistani rice, especially the Basmati cultivars, can provide best ingredients for functional foods.

## 1. Introduction

With the growing interest in functional foods and nutraceuticals and with a consequent expansion of health food market, there is much need to explore the profile of high-value components and functional bioactives of food crops [1,2]. Several cereal crops have been searched as a potential source of valuable bioactives including those of phenolics, tocopherols and oryzanol with multiple biological activities and medicinal functions [3–8]. In this context, introducing the varieties containing increased levels of such high-quality bioactive compounds may increase the commercial and nutritional value of the cereal crops [9].

The concentration of the bioactives and high-value components in natural food commodities such as cereals, fruits and vegetables vary with respect to species, varieties, the maturity stages as well as the agro-climatic conditions of the harvest [10–12]. The amount of such components is also affected due to post-harvest factors (storage conditions and processing) as well as due to changes in sample preparation and analysis regimes [13–16].

Rice (*Oryza sativa* L.) is recognized as the second most consumed staple food in the world, especially in Asian countries. In recent decades, unpolished rice has been searched as a rich source of important bioactive compounds and nutraceuticals with numerous potential health functions [17]. The major bioactives reported in the rice grain include the phenolic acids, polyphenols and ferulic esters (γ-oryzanol), tocopherols, tocotrienols and sterols [18–20].

Besides, rice has also received considerable recognition because of its healthy oil named as rice bran oil, extracted from the outer thin covering of the unpolished rice [21]. Information regarding different lipid components is essential for understanding of rice functionality. The contents of rice lipids may vary from 2.98 to 3.5% (hexane-extractable) [22]. Rice lipids contain 22–25% palmitic, 37–41% oleic acid and 37–41% linoleic acid [23]. Rice lipid phytosterols are considered to be vital natural compounds, because of their positive impacts on health. Experimental studies have proven that phytosterols reduce cholesterol levels of serum [24]. Rice has sufficient quantity of some tocopherols [25,26] which have potential physiological functionalities including cholesterol leveling and antithrombotic effects [27,28].

Similarly, another high-value component, known as γ-oryzanol with contribution in the range of 1.5–2.9% in rice oil, is regarded as a promising component for nutraceutical industry [29]. γ-oryzanol is more effective than tocopherols in imparting antioxidant activity to rice grain [30]. Chemically, this component is a mixture comprising steryl ferulates containing both esters of triterpene alcohols and plant sterols [31]. Cycloartenyl ferulate, 24-methylenecycloartanyl ferulate and campesteryl ferulate are recognized as the major components and account for 80 percent of γ-oryzanol [32]. The content of γ-oryzanol mostly depends upon rice grain variety, as long grain rice contain 6.42 mg/g and medium grain rice 5.17 mg/g [33].

Rice cultivated in different areas differs in grain shape, texture and flavor [34], suggesting possible differences in the composition of their bioactive nutrients. It has been established through studies that many rice varieties have differences in their physiochemical composition [35]. Although some reports have been documented on the nutrients profile of rice [36], however, to the best of our knowledge, no information is available in the literature on the detailed characterization of functional bioactives in commercial cultivars of Pakistani rice. The present research study was devised to quantify and compare the bioactives such as fatty acids, phytosterols, tocopherols and γ-oryzanol in 10 commercial Pakistani rice cultivars using state-of-the-art chromatographic tools such as GC, GC-MS and HPLC.

## 2. Results and Discussion

### 2.1. Rice Oil Yield

**Figure 1:**
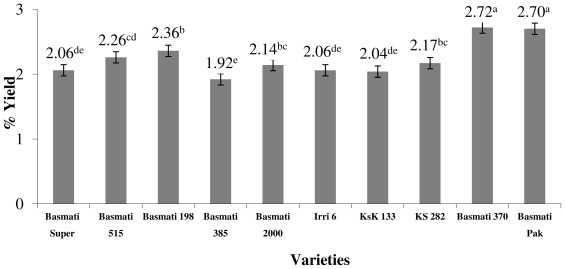
Oil yield (%) of various varieties of rice (*Oryza sativa* L). Means + standard deviations (*n =* 3); means followed by different letters in the bars differ significantly (*p* < 0.05) among varieties tested.

Rice contains considerable amount of oil with impressive functional food properties. In the present experiments, rice oils produced from the brown rice seed (unpolished rice), were evaluated for oil yields. The results in Figure 1 show that among rice varieties tested there is a significant (*p* < 0.05) difference observed for the amounts of crude oil. The hexane-extracted oil yield from the tested varieties of rice varied from 1.92 g/100g (Basmati 385) to 2.72 g/100 g (Basmati 370). In close agreement to the data of our analysis, Boonsit *et al.,* [37] also reported almost similar values, in the range of 2.00–3.07 g/100 g, for Malaysian rice. On the other hand, a study performed by Przybylski *et al.* [38] on North American wild rice (*Zizania palustris*), revealed the amounts of total lipids in the range of 0.7 to 1.1% showing that wild rice has considerably lower values as compared to regular rice (*Oryza sativa* L). Nutritionally, among the rice varieties tested in the present study, Basmati 370 offered the highest lipid yield.

### 2.2. Fatty Acid (FA) Contents of Rice

Rice oil samples, extracted using *n*-hexane were analyzed by gas liquid chromatography (GLC) after derivatizing into fatty acid methyl esters (FAMEs) and the data thus used to express the FA amounts on rice seed basis. The results presented in Table 1 depict the FA contents of different rice cultivars. There were seven saturated (C_14:0_, C_16:0_, C_18:0_) and unsaturated (C_18:1_, C_18:2_, C_18:3_, C_20:1_) FAs detected in the rice varieties tested. The contents of saturated fatty acids (SFA) including myristic (C_14:0_), palmitic (C_16:0_) and stearic (C_18:0_) acids in the rice ranged from 122–388 mg/kg, 3575–4961 mg/kg and 918–1602 mg/kg, respectively. The levels of unsaturated fatty acids (USFA) namely C_18:1_ (oleic acid), C_18:2_ (linoleic acid), C_18:3_ (linolenic acid) and C_20:1_ (gadoleic acid) ranged from 7042–10952 mg/kg, 5454–7028 mg/kg, 51–1550 mg/kg and 294–1177 mg/kg, respectively. It is evident that rice mainly contained oleic acid followed by linoleic acid. The composition of saturated and unsaturated fatty acids in different rice cultivars investigated in the present analysis closely resembled to that reported earlier by Anwar *et al.* [39] for four Basmati varieties of rice indigenous to Pakistan. The composition of SFA of the investigated varieties of rice was quite comparable with those of palm kernel, cottonseed and avocado seed oils [40]. The concentrations of major fatty acids C_18:2_, C_18:1_, C_18:0_, C_16:0_ of the investigated rice varieties were in close agreement with those reported by Hemavathy and Prabhakar [41] for the rice bran lipids from Indian based rice. However, the amount of C_16:0_ and C_18:0_ in the tested rice cultivars varied to some extent to those investigated by Hemavathy and Prabhakar [41]. The fatty acid composition of investigated rice resembled in the content of C_18:1_ with that of rice indigenous to Pakistan [16] however, varied to some extent with regard to other fatty acids.

The total contents, calculated on rice seed weight basis, of fatty acids, in the tested varieties of rice were highest in Basmati Pak (25840 mg/kg) while lowest in Basmati 370 (18,240 mg/kg). Lilitchan *et al.*, [42] investigated the total amount of fatty acids in rice of Thailand in the range of 18000–21000 mg/kg in rice, the values quite close to our present data. The total contents of fatty acids determined in the present work were also in line to those reported by Zhou *et al.* [43] in three different varieties of Australian rice (22,500–28,000 mg/kg).

### 2.3. Phytosterols Content of Rice

According to Piironen and Lampi [44], vegetable oils and their products are regarded as the rich natural sources of sterols, followed by cereal grains, cereal-based products and nuts. Plant sterols occur in cereals as free sterols, and in bound form as steryl esters of fatty acids and phenolic acids and glycosides. The phytosterol composition was analyzed using GC and GC-MS. β-Sitosterol with contribution varying from 446.2 mg/kg in Irri 6 to 656.4 mg/kg in Basmati Pak rice was established to be the major phytosterol component found in all the rice cultivars tested followed by campesterol, stigmasterol and Δ^5^-avenasterol. The total contents of campesterol, stigmasterol and Δ^5^-avenasterol in the rice cultivars ranged from 116.5–242.1 mg/kg, 75.3–181.0 mg/kg and 89.4–178.7 mg/kg. Cultivar Basmati Pak not only contained maximum content of β-sitosterol but also contained high amount of other three phytosterols. It is observed that Basmati Pak contained higher sterol contents relative to other cvs. (Table 2). Phytosterols play a positive role in reducing absorption of cholesterol and reducing the level of negative lipoproteins in human blood, thus potentially reducing the development of heart diseases [45]. The dietary intake of natural phytosterols ranges between 150–450 mg/day depending on eating style of a person [46]. In human diet β-sitosterol, campesterol and stigmasterol are the major and most common phytosterols distributed [47].

As far as the total amount of sterols is concerned, the highest level (1323.4 mg/kg) was found in Basmati Pak and the lowest (739.4 mg/kg) in KSK-133. No quantitative data for the total sterol contents of rice is reported as such with which we can compare our present values. Toivo *et al.* [48] reported total amount of rice sterols (7310 mg/kg of oil). Comparing the present rice phytosterol data (739.4–1323.4 mg/kg) with that of wheat having an amount of 600 mg/kg [48], it is understandable that rice varieties tested are a rich source of these valuable components.

In the present study, β-sitosterol, campesterol, stigmasterol and Δ^5^-avenasterol were found to be the main rice phytosterols which could be supported by some earlier studies. Kuroda *et al.* [49] isolated and analyzed sterols from rice bran oil and reported β-sitosterol, campesterol, and stigmasterol as predominant sterol components. Gaydou and Raonizafinimanana, [50] investigated Malagasy rice bran oils for sterol composition and found eight different sterols with β-sitosterol (53–59%), campesterol (16–26%) and stigmasterol (10–13%) as the predominant components. From the given discussion, it can be assumed that rice is a potential source of natural phytosterols to imparting health benefits. Vissers *et al.* [51] reported that 2.1 g sterols/day, derived from rice bran oil, can decrease LDL cholesterol by 9% and serum total cholesterol by 5% in normolipemic humans.

### 2.4. Tocopherols

Tocopherols provide protection to lipids against free radicals through their radical scavenging action [52]. Tocopherols, recognized as vitamin E isomers and having lipophilic property, are mainly present in the pericarp and endosperm part of cereal caryopses [53].

The composition of tocopherols [alpha-, gamma- and delta-tocopherols] in the tested varieties of rice was determined using high performance liquid chromatography (HPLC). The contents of different tocopherols, expressed on rice seed weight basis, in the selected varieties of rice is shown in Table 3. All the varieties tested revealed the presence of α-, γ- and δ- tocopherols. It is evident that concentration of α-tocopherol was comparatively higher than other isomers of tocopherols. The maximum value of γ-tocopherol (76.1 mg/kg of rice seed) was found in Basmati Pak and minimum (39.0 mg/kg of rice seed) in Irri-6. The content of γ-tocopherol in the selected varieties of rice was minimum (20.5 mg/kg of rice seed) in KS 282 and maximum (28.1 mg/kg of rice seed) in Basmati 198. The analysis revealed the amount of δ-tocopherol to be the highest in Basmati 370 (16.5 mg/kg of rice seed) while the lowest in Irri6 (6.5 mg/kg of rice seed). The total tocopherol content comprising α-, γ- and δ-tocopherols ranged from 67.1 mg/kg of rice seed (Irri6) to 115.3 mg/kg of rice seed (Basmati Pak). Basmati Pak was found to be the best variety regarding the amount of tocopherols. The selected varieties of rice were found to be a potential source of α-, γ- and δ-tocopherols whereas β-tocopherol was not detected. It is widely accepted that δ-tocopherol has greater antioxidant potency than that of γ- or α-tocopherol [54]. Yawadio *et al.* [55] studied the black pigmented rice and detected four isomers of tocopherols (α-, β-, γ-, δ-) with total contents as70 mg/kg. Rice bran contains over 300 mg/kg of vitamin E [23].

Rice (*Oryza sativa*) is one of the rich natural sources of vitamin E like tocopherols and tocotrienols, containing wholesome amount up to levels as high as 300 mg/kg [23]. Approximately 1.0% of the unsaponifiable fraction of rice oil is α-tocopherol. Studies show that 1.0 g of oil contains 3.0 mg of α-tocopherol [56]. Due to presence of considerable amounts of natural antioxidants such as tocopherols, rice oil is worthwhile to improving the storage and frying stability of oils [57]. Ha *et al.* [58] examined the effect of degree of milling on tocopherols in rice and found that total tocopherol contents of rice samples after milling were very low (37.7 mg/kg) indicating that the removal of the hull and milling process reduced the content of tocopherols.

### 2.5. γ-Oryzanol

γ-Oryzanol, a commonly found bioactive component in rice, is rarely found in other cereals and vegetables. Generally, crude rice oil contains more than 2% unsaponifiable matter comprising mainly γ-oryzanol along with phytosterols and tocopherols *etc.* [59]. The antioxidant activity of γ-oryzanol can be attributed to its structure, which includes ferulic acid with strong antioxidant activity [32,56,60]. Rice bran oil contains about 3000 mg/kg of γ-oryzanol, which is a mixture of several components of ferulate esters of triterpene alcohol [61].

γ-Oryzanol contents in the tested varieties of rice were analyzed rice lipids using HPLC and calculations made on rice seed weight basis Table 4. The composition of γ-oryzanol (mg/kg of brown seed rice) of different varieties of rice revealed the presence of four different components of γ-oryzanol including those of cycloartenyl ferulate, 24-methylene cycloartanyl ferulate, campesteryl ferulate and β-sitosteryl ferulate. The amounts of these components mostly varied (*p* < 0.05) among selected varieties of rice. The levels of principal component, 24-methylene cycloartanyl ferulate, among the rice varieties tested varied from 140.8–183.1 mg/kg. The content of second major compound *i.e*. cycloartenyl ferulate was found to be maximum (103.6 mg/kg) in Basmati Pak and minimum (65.5 mg/kg) in KSK-133. Campesteryl ferulate, the third main component detected, ranged from 29–45 mg/kg, while the least prevalent compound namely β-sitosteryl ferulate was 8.56–10.42 mg/kg. Overall, the contents of the detected γ-oryzanol components were higher in the Basmati varieties of rice (Basmati Pak, Basmati Super and Basmati 370) revealing their higher antioxidant and functional food potential. The other varieties tested also contained considerable levels of γ-oryzanol. The total amounts (mg/kg of rice seed) of γ-oryzanol among varieties of rice tested varied from 246.7–330.3 mg/kg. Basmati Pak was found to be the best variety with regard to the amount of γ-oryzanol.

Several earlier studies support our present data on rice γ-oryzanol. Xu and Godber, [62] extracted γ-oryzanol from rice yielding a maximum of 1.68 mg/g of rice. Norton, [63] reported that the oryzanol group is unique in rice and the exact composition of oryzanol depends on rice cultivars. Almost 10 different fractions of γ-oryzanol isomers from rice have been successfully identified and isolated using a reverse-phase HPLC [64]. According to some reports, the contents of γ-oryzanol (115–780 mg/kg) differ with the source of rice, depending on the degree of processing [31]. Azrina *et al.* [65] analyzed oryzanol in rice and the values determined were in agreement with those of the present study. The rice processing methods may affect oryzanol contents and a major part of this valuable compound is lost during the oil refining process [21].

Overall, on the basis of the amounts of high-value bioactive components investigated such as essential fatty acids, tocopherols, phytosterols and γ-oryzanol, the Basmati varieties, especially Basmati Pak was established to be relatively the superior variety among others, suggesting its potential uses as ingredients of functional foods and nutraceuticals to benefit health of consumers and promoting value-addition.

## 3. Experimental Section

### 3.1. Collection of Rice (*Oryza sativa* L.*)* Samples

Samples of ten commercial varieties namely Basmati Super, Basmati 515, Basmati 198, Basmati 385, Basmati 2000, Basmati 370, Basmati Pak, KS 133, KSK 282 and Irri 6 of paddy rice (*Oryza sativa* L.) were procured from the Rice Research Institute, Kalashahkako, Lahore, Pakistan. Three different samples for each of the rice cultivar cultivated at different locations of the rice farms were harvested and pooled. Samples from fully ripened crop were taken. For each variety a sample of 5.0 kg was collected from fields grown in the same environment.

### 3.2. Rice Lipids Extraction

Lipids from all ten rice varieties were extracted with *n*-hexane using Soxhelt extraction apparatus. Briefly, the ground rice (100 g) material (80 mesh size) was separately filled into a thimble and placed into a Soxhlet assembly fitted with a 500 mL round-bottom flask and a water condenser. The extraction was performed on a controlled heating assembly (Behr, Labor-Tecknik) for 6 h. After extraction, excess of the solvent was then distilled off using a vacuum rotary evaporator (EYELA, N-N Series, Rikakikai Co., Ltd.) at 45 °C.

### 3.3. Fatty Acids Composition

Fatty acids compositional (FAs) analysis of the extracted rice oil (RO) was performed using a capillary gas chromatography. The standard IUPAC method 2.301 was used to prepare fatty acid methyl esters through *trans*-esterification [66]. FAMEs were analyzed using a gas chromatograph (Shimadzu GC 17A), fitted with a SP-2330, methyl-lignocerate-coated (film thickness = 0.20 μm) polar capillary column (30 m × 0.32 mm), and a flame ionization detector (FID). The column oven temperature was initially held at 160 °C for 1 min and raised to 220 °C by the rate of 5 °C per min and finally held for 10 min. Nitrogen was used as a carrier gas at a flow rate 3 mL/min. The injector and detector were set at 230 and 250 °C, The unknown FAMEs were then identified using reference standards from Sigma-Aldrich Chemical Co., (St. Louis, MO, USA). Fatty acid composition was reported as a relative percentage of the total peak area.

### 3.4. Sterol Composition

#### 3.4.1. Saponification

For sterol analysis, the procedure reported by [48] with slight modifications was used. Briefly, 50 mg oil sample was weighed into a 50 mL round-bottom flask, added 5 mL of 0.5 M methanolic KOH and the sample refluxed for 30 min at 80 °C. After the saponification step, the unsaponifiable material recovered was spiked with an aliquot of internal standard solution (1.0 mg 5-α-cholestane).

#### 3.4.2. Solid-Phase Extraction and Purification of Sterol Fraction

An aliquot of 2–3 mL taken from the chloroform layer of the solution was transferred into a test tube followed by addition of a few drops of 5 M HCL to acidify the solution (pH 2.5). One milliliter of the pH-adjusted solution was allowed to pass through a 0.45 mm nylon membrane filter (Whatman) onto the pre-activated C_18_ solid-phase extraction cartridge. The multi sets of cartridges were sequentially activated using 5 mL methanol and 5 mL deionized water. The sample solutions were eluted in drop-wise manner. The elution of the sterol fraction was performed using 15 mL of solution mixture containing 5% methanol in chloroform. The recovered eluates, with purified sterol fraction, were further concentrated to volume as low as 0.5 mL.

#### 3.4.3. Silylation

An aliquot (100–200 mL) of solution was transferred into a sample vial and evaporated to dryness under nitrogen streaming. The residue obtained was dissolved in 100 μL anhydrous pyridine. Trimethylsilyl (TMS) derivatives were prepared by adding 100 μL of the derivatizing reagent comprising bistrifluoroacetamide (BSTFA 99%) and trimethylchlorosilane (TMCS 1%). The solutions were allowed to react over-night (room temperature) to complete the silylation process. The silyl-derivatives of sterols were analyzed by GC using a Perkin Elmer system fitted with a methyl phenyl polysiloxanes coated capillary column OV-17 (30 m × 0.25 mm, 0.20 μm film thicknesses) and a flame ionization detector (FID). The column was isothermally operated at temperature of 260 °C. Injector and FID temperatures were set at 275 and 290 °C, respectively. Extra pure N_2_ at a flow rate of 3.5 mL/min was used as a carrier gas. Identification and quantification of unknown sterol components was done using a pure mixture (>95%) of sterol standards (Sigma-Aldrich Chemical Co., St. Louis, MO, USA). For authentification purposes, the samples were also analyzed using GC-MS under the same column chromatographic conditions as specified for GC analysis. An Agilent-Technologies (Little Falls, CA, USA) 6890 N Network GC system, fitted with an inert XL Mass selective detector (Agilent-Technologies 5975) and auto-injector (Agilent-Technologies 7683B series) was used. For sterol components detection, an electron ionization (EI) mode with ionization energy 70 eV was employed while the injector and MS transfer line temperatures were maintained at 275 and 290 °C, respectively. Scanning mass range was selected from 50–600 m/z. The sterol compounds were identified by comparing their relative and absolute retention times with those of authentic standards of sterols. The target compounds were further identified and authenticated using their MS spectral information compared to those from the mass spectral library (NIST) of the GC/MS system. The calculations were made on rice grain weight basis.

### 3.5. Tocopherol Composition

Tocopherols (α, β, γ and δ) in the rice bran oils were analyzed by RP-HPLC (Sykam GmbH, Kleinostheim, Germany) according to the method as described by [32] with some modifications. The HPLC instrument used was equipped with S-1122 dual piston solvent delivery system. Briefly, rice oil (100 mg) was dissolved into 5 mL methanolic KOH (80%) and subjected to incubation at 65 °C for 30 min. After the saponification was completed, the mixture was allowed to cool down to room temperature. For extraction purposes, 3 mL distilled water and 10 mL *n*-hexane were added to the sample. The hexane layer recovered, containing tocopherols components, was evaporated to dryness under nitrogen streaming and then added 1.0 mL mobile phase to re-dissolve the residue. Tocopherol isomers were separated on a Hypersil ODS reverse phase (C_18_) column (250 × 4.6 mm, 5 μm particle size; Thermo Hypersil GmbH, Darmstadk, Germany) fitted with a C_18_ guard column. The mobile phase used was a mixture of methanol: acetonitrile: dichloromethane (50:44:6, v/v/v) at a flow rate of 1.0 mL/min. The detection was performed at 295 nm using S-3210 UV/VIS diode array detector (DAD). Tocopherol isomers were identified on the basis of matching of their retention times with those of pure (≥96%) standards (Sigma-Aldrich Chemical Co., St. Louis, MO, USA) of tocopherols and quantified on the basis of peak area using standard calibration curve of the pure compounds.

### 3.6. Oryzanol Composition

Gamma-oryzanol content in oils of different varieties of rice was also determined using reverse phase high performance liquid chromatography (RP-HPLC) according to the method reported by [31] with slight modifications. Rice oil (100 mg) was dissolved in 1.0 mL of pure methanol and then filtered using a syringe filter with PTFE (0.2 μm; Ascordic syringe filter). A RP-HPLC system **(**SEL 10AL, Shimadzu, Japan) equipped with Hypersil ODS reverse phase (C_18_) column (250 × 4.6 mm, 5 μm particle size; Thermo Hypersil GmbH, Darmstadk, Germany) and a variable wavelength UV-Vis detector (SPD 10A, Shimadzu, Kyoto, Japan) set at 330 nm was used. Mobile phase consisting of a mixture of methanol: acetonitrile: dichloromethane: acetic acid (50:44:3:3 v/v/v/v) at a flow rate of (isocratic) of 1.0 mL/min was used. The total analysis time was approximately 22 min and γ-oryzanol peaks appeared around retention time of 9–15 min. Gamma-oryzanol content (mg/kg) in the rice oil was calculated from the peak area of standard γ-oryzanol calibration curve. The identification was based on the comparison of the retention times of the unknown with those of pure (>95%) standards of gamma-oryzanol (Tokyo Chemical Company, Tokyo, Japan).

### 3.7. Statistical Analysis

Three different samples of rice for each variety were assayed. Each sample was analyzed individually in triplicate and data is reported as mean (*n* = 3 × 3) ± SD (*n* = 3 × 3). ANOVA was used to determine significant differences considering a level of significance at less than 5% (*p* < 0.05) by using the statistical software Co-Stat (version 6.3; Stat Soft Inc, Tulsa, OK, USA, 2004).

## 4. Conclusions

On the basis of these valuable components including fatty acids, phytosterols, tocopherols and γ-oryzanol, Basmati Pak rice was found to be the superior variety having total amount of fatty acids 25,840 mg/kg (Table 1), total phytosterols content (1323.4 mg/kg) (Table 2), total tocopherols content 115.5 mg/kg and γ-oryzanol contents 330.3 mg/kg.

Overall, the tested varieties of Pakistani rice, and especially Basmati cultivars, are a good source of valuable nutrients and high-value components such as tocopherols, phytosterols and γ-oryzanol, which suggests the use of these rice cultivars for functional food and nutraceutical application. Besides, it is also evident that variations may exist for the distribution of total bioactives/nutrients in relation to the rice genotype, as well as a function of extraction system used for the isolation of such components. Therefore, comprehensive studies on the isolation, purification and structural elucidation of some novel rice bioactives/phenolics coupled with activity-directed evaluation of these compounds for specific functional food application are strongly recommended.

## Figures and Tables

**Table 1:** Fatty acid contents (mg/kg) of brown rice seed (unpolished rice) of different varieties of rice (*Oryza sativa* L.).

Varieties	C ^14:0^	C ^16:0^	C ^18:0^	C ^18:1^	C ^18:2^	C ^18:3^	C ^20:1^	Total
**Basmati Super**	156 ± 8 ^c^	3613 ± 224 ^c^	918 ± 65 ^d^	7207 ± 360 ^cd^	6211 ± 310 ^ab^	1063 ± 53 ^c^	352 ± 17 ^g^	19520 ± 976 ^c^
**Basmati 515**	150 ± 7 ^d^	4216 ± 274 ^bc^	1198 ± 53 ^b^	7939 ± 396 ^bcd^	5971 ± 298 ^de^	749 ± 37 ^d^	1177 ± 59 ^a^	21400 ± 1070 ^bc^
**Basmati 198**	179 ± 9 ^c^	4484 ± 205 ^ab^	1300 ± 49 ^b^	7892 ± 394 ^d^	6569 ± 328 ^cde^	1256 ± 63 ^c^	740 ± 37 ^c^	22420 ± 1121 ^b^
**Basmati 385**	205 ± 11 ^c^	5489 ± 205 ^a^	1077 ± 48 ^e^	10952 ± 547 ^a^	7875 ± 393 ^bc^	51 ± 3 ^f^	-	25650 ± 1285 ^a^
**Basmati 2000**	122 ± 6 ^e^	4107 ± 189 ^ab^	996 ± 48 ^cd^	8417 ± 420 ^a^	6201 ± 310 ^bc^	61 ± 3 ^f^	427 ± 21 ^f^	20330 ± 1016 ^bc^
**Irri 6**	137 ± 7 ^d^	3797 ± 189 ^bc^	978 ± 55 ^cd^	7495 ± 374 ^bc^	6497 ± 324 ^a^	372 ± 19 ^e^	294 ± 14 ^h^	19570 ± 980 ^c^
**KSK 133**	213 ± 11 ^b^	3954 ± 197 ^ab^	969 ± 52 ^c^	6841 ± 342 ^d^	5620 ± 281 ^cde^	1143 ± 57 ^b^	640 ± 32 ^d^	19380 ± 965 ^c^
**KS 282**	140 ± 7 ^d^	3990 ± 199 ^ab^	1117 ± 80 ^b^	7042 ± 352 ^b^	5925 ± 296 ^d^	1197 ± 60 ^bcd^	539 ± 27 ^e^	19950 ± 990 ^bc^
**Basmati 370**	128 ± 6 ^d^	3575 ± 178 ^bc^	1058 ± 71 ^b^	7278 ± 363 ^ab^	5454 ± 272 ^bcd^	310 ± 15 ^e^	438 ± 22 ^e^	18240 ± 912 ^c^
**Basmati Pak**	388 ± 20 ^a^	4961 ± 248 ^bc^	1602 ± 95 ^a^	9328 ± 466 ^cd^	7028 ± 351 ^e^	1550 ± 77 ^a^	982 ± 50 ^b^	25840 ± 1292 ^a^

Values are mean ± SD for three samples of each variety, analyzed individually in triplicate (*n* = 3 × 3). Means with different superscript letters within the same column indicate significant differences (*p* < 0.05) among varieties tested.

**Table 2:** Phytosterol contents (mg/kg) of brown rice seed (unpolished rice) of different varieties of rice (*Oryza sativa* L.).

Varieties	Stigmasterol	Stigmastanol	β-Sitosterol	Campesterol	Δ^5^-avenasterol	Δ^7^-avenasterol	Total
**Basmati Super**	94.8 ± 4.8 ^e^	31.7 ± 1.5 ^e^	489.6 ± 24.4 ^bcd^	172.3 ± 7.6 ^b^	152.7 ± 7.6 ^d^	51.6 ± 2.6 ^e^	905.7 ± 45 ^de^
**Basmati 515**	148.5 ± 7.4 ^fg^	48.0 ± 2.4 ^g^	493.2 ± 25.0 ^efg^	161.4 ± 8.9 ^c^	178.7 ± 8.9 ^d^	50.0 ± 2.5 ^d^	932.7 ± 46 ^d^
**Basmati 198**	151.7 ± 7.6 ^b^	33.2 ± 1.6 ^d^	584.9 ± 29.1 ^ab^	186.2 ± 7.2 ^b^	144.7 ± 7.2 ^b^	42.2 ± 2.1 ^a^	1142.7 ± 58 ^c^
**Basmati 385**	158.3 ± 8.0 ^gh^	65.7 ± 3.2 ^c^	590.9 ± 23.7 ^fg^	210.7 ± 9.1 ^d^	183.7 ± 9.2 ^c^	71.0 ± 3.5 ^d^	1050.6 ± 52 ^c^
**Basmati 2000**	134.0 ± 6.7 ^a^	21.9 ± 1.1 ^b^	473.4 ± 22.3 ^a^	162.6 ± 5.8 ^a^	117.4 ± 5.8 ^a^	31.1 ± 1.5 ^b^	1040.0 ± 53 ^c^
**Irri 6**	75.3 ± 3.5 ^h^	73.9 ± 3.7 ^h^	446.2 ± 23.6 ^g^	144.9 ± 6.1 ^e^	123.4 ± 6.1 ^e^	82.2 ± 4.1 ^f^	739.4 ± 37 ^f^
**KSK 133**	124.8 ± 6.2 ^ef^	69.5 ± 3.4 ^g^	472.2 ± 24.0 ^cde^	116.5 ± 5.3 ^d^	89.4 ± 4.4 ^e^	67.4 ± 3.3 ^e^	827.2 ± 41 ^e^
**KS 282**	154.9 ± 7.7 ^d^	56.0 ± 2.8 ^f^	478.1 ± 22.2 ^def^	139.2 ± 7.3 ^d^	102.3 ± 5.1 ^d^	60.0 ± 3.0 ^e^	853.9 ± 42 ^de^
**Basmati 370**	160.3 ± 8.0 ^c^	19.1 ± 1.0 ^c^	445.4 ± 21.3 ^cde^	154.6 ± 9.2 ^b^	108.4 ± 5.4 ^b^	45.8 ± 2.3 ^c^	865.8 ± 44 ^de^
**Basmati Pak**	181.0 ± 9.1 ^b^	34.0 ± 1.7 ^a^	656.3 ± 32.8 ^abc^	242.1 ± 12.1 ^a^	174.8 ± 8.7 ^a^	36.1 ± 1.8 ^cd^	1323.4 ± 66 ^a^

Values are mean ± SD for three samples of each variety, analyzed individually in triplicate (*n* = 3 × 3). Means with different superscript letters within the same column indicate significant differences (*p* < 0.05) among varieties tested.

**Table 3:** Tocopherols (mg/kg) of brown rice seed (unpolished rice) of different varieties of rice (*Oryza sativa* L.).

Varieties	α-Tocopherol	γ-Tocopherol	δ-Tocopherol	Total
**Basmati Super**	72.3 ± 2.8 ^ab^	27.5 ± 1.2 ^ab^	12.1 ± 0.6 ^c^	111.9 ± 6 ^a^
**Basmati 515**	58.2 ± 2.4 ^c^	25.6 ± 1.3 ^bc^	10.5 ± 0.5 ^e^	94.3 ± 5 ^b^
**Basmati 198**	69.0 ± 3.2 ^b^	28.1 ± 1.4 ^a^	11.5 ± 0.5 ^cd^	108.6 ± 5 ^a^
**Basmati 385**	61.2 ± 2.4 ^c^	22.5 ± 1.1 ^de^	8.5 ± 0.3 ^f^	92.2 ± 4 ^b^
**Basmati 2000**	57.2 ± 2.1 ^c^	27.5 ± 1.3 ^ab^	10.7 ± 0.5 ^de^	95.4 ± 4 ^b^
**Irri 6**	39.0 ± 2.0 ^f^	21.6 ± 1.0 ^e^	6.5 ± 0.3 ^g^	67.1 ± 3 ^d^
**KSK 133**	41.2 ± 1.9 ^ef^	24.2 ± 1.2 ^cd^	9.0 ± 0.4 ^f^	74.4 ± 3 ^c^
**KS 282**	45.2 ± 2.0 ^e^	20.5 ± 1.0 ^e^	14.2 ± 0.6 ^b^	79.9 ± 4 ^c^
**Basmati 370**	51.2 ± 2.7 ^d^	26.7 ± 1.3 ^ab^	16.5 ± 0.5 ^a^	94.4 ± 4 ^b^
**Basmati Pak**	76.1 ± 3.1 ^a^	25.75 ± 1.2 ^bc^	13.7 ± 0.6 ^b^	115.5 ± 6 ^a^

Values are mean ± SD for three samples of each variety, analyzed individually in triplicate (*n* = 3 × 3). Means with different superscript letters within the same column indicate significant differences (*p* < 0.05) among varieties tested.

**Table 4:** γ-Oryzanol content (mg/kg) of brown rice seed (unpolished rice) of different varieties of rice (*Oryza sativa* L.).

Varieties	Cycloartenyl Ferulate	24-Methylene Cycloartanyl Ferulate	Campesteryl Ferulate	β-Sitosteryl Ferulate	Total
**Basmati Super**	82.2 ± 2.1 ^d^	174.3 ± 3.4 ^b^	31.0 ± 0.3 ^f^	10.2 ± 0.3 ^f^	297.7 ± 15 ^bcd^
**Basmati 515**	92.4 ± 2.3 ^b^	164.1 ± 2.7 ^c^	41.4 ± 0.4 ^c^	9.8 ± 0.4 ^c^	307.7 ± 15 ^ab^
**Basmati 198**	83.5 ± 2.4 ^d^	144.3 ± 2.5 ^ef^	45.5 ± 0.3 ^a^	9.5 ± 0.5 ^a^	282.8 ± 14 ^cd^
**Basmati 385**	80.0 ± 1.9 ^d^	154.9 ± 3.1 ^d^	32.2 ± 0.5 ^e^	10.4 ± 0.4 ^e^	277.5 ± 13 ^de^
**Basmati 2000**	88.5 ± 2.0 ^c^	147.1 ± 3.2 ^e^	36.6 ± 0.8 ^d^	10.1 ± 0.3 ^d^	282.3 ± 14 ^cd^
**Irri 6**	83.1 ± 2.1 ^c^	183.1 ± 2.3 ^a^	29.8 ± 0.3 ^g^	8.6 ± 0.4 ^g^	304.6 ± 15 ^bc^
**KSK 133**	65.5 ± 1.5 ^f^	140.2 ± 2.6 ^f^	31.5 ± 0.4 ^ef^	9.5 ± 0.5 ^ef^	246.7 ± 12 ^f^
**KS 282**	72.0 ± 1.3 ^e^	142.9 ± 2.8 ^ef^	30.1 ± 0.5 ^g^	8.6 ± 0.3 ^g^	253.6 ± 12 ^ef^
**Basmati 370**	92.3 ± 2.1 ^b^	166.2 ± 3.1 ^c^	44.4 ± 0.6 ^b^	9.2 ± 0.4 ^b^	312.1 ± 15 ^ab^
**Basmati Pak**	103.6 ± 2.7 ^a^	171.7 ± 2.8 ^b^	45.2 ± 0.5 ^ab^	9.8 ± 0.5 ^ab^	330.3 ± 17 ^a^

Values are mean ± SD for three samples of each variety, analyzed individually in triplicate (*n* = 3 × 3). Means with different superscript letters within the same column indicate significant differences (*p* < 0.05) among varieties tested.
